# Serum citrullinated histone H3 concentrations differentiate patients with septic verses non-septic shock and correlate with disease severity

**DOI:** 10.1007/s15010-020-01528-y

**Published:** 2020-09-30

**Authors:** Yuzi Tian, Rachel M. Russo, Yongqing Li, Monita Karmakar, Baoling Liu, Michael A. Puskarich, Alan E. Jones, Kathleen A. Stringer, Theodore J. Standiford, Hasan B. Alam

**Affiliations:** 1 Department of Surgery, University of Michigan Health System, University of Michigan Medical School, 1500 E Medical Center Dr. SPC 5331, Ann Arbor, MI 48109-5331 USA; 2 Department of Rheumatology and Immunology, Xiangya Hospital, Central South University, Changsha, Hunan China; 3 Department of Emergency Medicine, Hennepin County Medical Center, Minneapolis, MN USA; 4 Department of Emergency Medicine, University of Minnesota, Minneapolis, MN USA; 5 Department of Emergency Medicine, University of Mississippi Medical Center, Jackson, MS USA; 6 Department of Clinical Pharmacy, College of Pharmacy, University of Michigan, Ann Arbor, MI USA; 7 Division of Pulmonary and Critical Care Medicine, Department of Internal Medicine, University of Michigan School of Medicine, Ann Arbor, MI USA

**Keywords:** Sepsis, Citrullinated histone H3, Peptidylarginine deiminase, Outcomes, Diagnosis

## Abstract

**Purpose:**

Microbial infection stimulates neutrophil/macrophage/monocyte extracellular trap formation, which leads to the release of citrullinated histone H3 (CitH3) catalyzed by peptidylarginine deiminase (PAD) 2 and 4. Understanding these molecular mechanisms in the pathogenesis of septic shock will be an important next step for developing novel diagnostic and treatment modalities. We sought to determine the expression of CitH3 in patients with septic shock, and to correlate CitH3 levels with PAD2/PAD4 and clinically relevant outcomes.

**Methods:**

Levels of CitH3 were measured in serum samples of 160 critically ill patients with septic and non-septic shock, and healthy volunteers. Analyses of clinical and laboratory characteristics of patients were conducted.

**Results:**

Levels of circulating CitH3 at enrollment were significantly increased in septic shock patients (*n* = 102) compared to patients hospitalized with non-infectious shock (NIC) (*n* = 32, *p* < 0.0001). The area under the curve (95% CI) for distinguishing septic shock from NIC using CitH3 was 0.76 (0.65–0.86). CitH3 was positively correlated with PAD2 and PAD4 concentrations and Sequential Organ Failure Assessment Scores [total score (*r* = 0.36, *p* < 0.0001)]. The serum levels of CitH3 at 24 h (*p* < 0.01) and 48 h (*p* < 0.05) were significantly higher in the septic patients that did not survive.

**Conclusion:**

CitH3 is increased in patients with septic shock. Its serum concentrations correlate with disease severity and prognosis, which may yield vital insights into the pathophysiology of sepsis.

**Electronic supplementary material:**

The online version of this article (10.1007/s15010-020-01528-y) contains supplementary material, which is available to authorized users.

## Introduction

Sepsis remains a leading cause of mortality, despite advances in antibiotic treatment, resuscitation, and supportive care. Early diagnosis and prompt initiation of treatments (including antibiotics) are essential for improving outcomes. However, indiscriminate administration of broad-spectrum antibiotics may increase bacterial resistance to commonly used agents. Improving the rapid identification and accurate treatment of sepsis is facilitated by identifying key pathways in its pathogenesis [1].

Systemic microbial infection induces immune cell activation and death. DNA and cellular proteins such as elastase, myeloperoxidase, and histones (particularly histone H3) are released into the extracellular milieu. These substrates form a net-like structure that can trap invading pathogens [2, 3]. Such extracellular traps (ETs), including neutrophil extracellular traps (NETs) and macrophage/monocyte extracellular traps (METs), are effective in killing bacteria but may cause damage to the host as well [3, 4]. Excessive NETosis/METosis has been implicated in the development of the clinical manifestations of sepsis [3, 5].

PADs are a family of calcium-dependent enzymes that regulate immune activity through a process known as citrullination [6]. Among them, PAD2 and PAD4 are involved in the signaling pathway of NETosis/METosis [7, 8]. Meanwhile, PAD2 induces inflammatory changes important in sepsis such as enhanced microvascular permeability and increased cytokine production.

The citrullination of histone 3 (CitH3) catalyzed by PAD2 alters gene expression and protein transcription in a manner that may be specific to the sepsis response [9]. We have identified elevated levels of serum CitH3 in mice subjected to endotoxic and septic shock but not in those subjected to hemorrhagic shock [10, 11]. Additionally, CitH3 released during NETosis serves as a damage-associated molecular pattern (DAMP) that induces a positive feedback loop [12]. CitH3 then stimulates perpetual NETosis in response to on-going infection, increasing serum concentrations in mice in the absence of appropriate antimicrobial therapy. Understanding the molecular mechanisms of PADs and CitH3 in the pathogenesis of septic shock in humans will be an important next step to developing novel diagnostic and treatment modalities.

In this study, we sought to determine the expression of CitH3 in patients with septic shock, and to correlate CitH3 levels with PAD2, PAD4 and clinically relevant outcomes.

## Methods

### Study design

This is a retrospective analysis of prospectively collected data and serum samples obtained during a consecutive enrollment, two-site (University of Mississippi Medical Center and University of Michigan) observational cohort study that was approved by each center’s institutional review board (IRB#201-0261 and HUM00056630, respectively). All patients or their legal representative gave written informed consent for enrollment. Septic shock patients (SP) were adults (≥ 18 years) presenting to their respective emergency departments (ED) who met the consensus definitions for septic shock during the period of enrollment: confirmed or suspected infection, two or more systemic inflammatory response criteria [13], and hypo-perfusion evidenced by either a systolic blood pressure of < 90 mmHg after fluid resuscitation or a blood lactate level of at least 36 mg/dL [14]. Non-infectious controls (NIC) were patients who presented to the emergency department in shock, as defined as either the need for vasopressors or persistent hypotension (SBP < 90, MAP < 65) after 2 L of fluid resuscitation, and without evidence of infection. SP and NIC were matched for age (± 5 years), sex, and date of presentation (± 7 days). Health volunteers (HVs) were ambulatory non-smokers under 60 years of age, who had no known chronic medical condition, were taking no medications, and were asymptomatic at the time of enrollment. Exclusion criteria included (1) inability to provide informed consent; (2) transferred from another hospital setting with therapy initiated; (3) do not resuscitate status or advance directives restricting illness-specific aggressive care or if the treating physician deemed aggressive care unsuitable; (4) cardiopulmonary resuscitation (chest compression or defibrillation) prior to enrollment.

All patients received standardized fluid resuscitation, which was initiated in the ED and continued during hospitalization, either in the intensive care unit (ICU) or non-ICU acute care unit. Clinical and outcomes data were collected throughout hospitalization, and the 90-day follow-up period. Data collection was performed by trained research assistants. All-cause mortality was assessed at 90 days and was cross-referenced with the US Social Security Death Index for verification.

### Blood sample analysis

Blood samples were collected at enrollment in all subjects, and then at 24 h, and 48 h following enrollment in septic shock patients that remained alive and hospitalized. Blood was drawn into a serum-separating tube, was allowed to clot at least 30 min, and was subsequently centrifuged (10 min at 1800 × g at 15 °C) at which time serum was aliquoted in 0.5 mL increments and stored (− 80 °C) until the time of assay. Samples collected at the University of Mississippi were mailed in batches on dry ice to the University of Michigan, where they were transferred to  − 80 ^O^C until the time of assay.

Quantification of CitH3 was performed with a sandwich enzyme-linked immunosorbent assay (ELISA) we developed in house; details have been previously published [10]. This was done because the commercially available anti-CitH3 antibodies only recognize areas of peptidylargine deiminase (PAD) 4 citrullination (R2 + R8 + R17) on the CitH3 protein, PAD2 catalyzed citrullination sites may not be detected (R26) [15]. To circumvent these problems, we used our proprietary anti-histone H3 (citrullinated R2 + R8 + R17 + R26) antibody to quantify CitH3 [12]. The person performing the assay was blinded to the group allocation of the serum samples.

Procalcitonin (PCT), PAD2, and PAD4 levels were measured using commercial ELISA kits (PCT: DY8350-05, R&D Systems Inc., Minneapolis, MN, USA; PAD2: Cayman Chemical #501450; PAD4: Cayman Chemical #501460). All procedures were performed in accordance with the manufacturer’s instructions.

#### Clinical and laboratory data collection

The comprehensive clinical and laboratory data obtained during the hospitalization were those recorded in the study’s RedCap data base, including age, gender, ventilation, hospital stay length, SOFA score, lactate, and 90 days survival. To further examine the relationship between the CitH3 levels and clinical features, extra clinical parameters were obtained through individual chart review at the University of Michigan, including temperature, white blood cell count (WBC), neutrophil counts, discharge diagnosis, culture data, and underlying comorbidities including immunosuppression. Patients were categorized as immunosuppressed if they met any of the following conditions: (1) chronic use of immunosuppressive drugs; (2) a current diagnosis of malignancy, autoimmune or inflammatory bowel disease for which they were prescribed immunosuppressive drugs; (3) acquired immunity deficiency syndrome. This review of the medical records was approved by the Institutional Review Board at the University of Michigan.

### Statistical analysis

Categorical values were described using numbers and percentages, and continuous variables were described using means and standard deviations or medians and interquartile ranges based on distribution of the data. A Kruskal–Wallis test followed by Dunn’s multiple comparisons test was performed to determine if there were significant differences in CitH3 and PCT levels in the three groups and three time points. The effect of time on CitH3 and PCT levels in patients admitted with septic shock was also analyzed using a linear mixed effects regression model. The receiver operating characteristic curves (ROC) were constructed to test the diagnostic and prognostic accuracy of CitH3 and PCT, the optimal cut-off values were determined using the Youden index to maximize the sum of sensitivity and specificity. The sensitivity, specificity, positive predictive value, and negative predictive value were calculated based on the optimal cut-off value. The equality of the ROC curves for CitH3 or for PCT was formally tested using Wald Statistic [16]. Nonparametric data between survivors and non-survivors were compared with the Mann–Whitney *U* test. Correlation between CitH3 and PAD2, PAD4, total SOFA score, individual SOFA component scores, and serum lactate values at the ED enrollment were analyzed using Pearson correlation model.

In a sub-group of the University of Michigan cohort, post hoc analysis was performed using discharge diagnosis to re-categorize patients into groups based on the presence or absence of an identified source of microbial infection (Supplemental Table 1). The AUCs of CitH3 and PCT to distinguish septic shock patients with an identified infection source (PIS) and those with un-identified infection source (PUIS) were compared. Descriptive analysis of available culture data was performed. Correlation between CitH3 and immunosuppression state, temperature, white blood cell, and neutrophil counts values at the ED enrollment were analyzed using Pearson correlation model.

All analyses and figures were conducted and drawn using GraphPad Prism (San Diego, CA, USA) or STATA v15.2 (StataCorp, College Station, TX, USA). For all analyses*,* *p* < 0.05 was considered statistically significant, and 95% CI were presented as appropriate.

## Results

### Baseline characteristics

A total of 160 subjects were enrolled from two hospitals (Supplemental Fig 1), including 89 [64 septic shock patients (SP); 25 patients hospitalized with non-infectious shock that served as controls (NIC); 19 health volunteers (HV)] from the University of Michigan (UMich) from May to July in 2012, and 52 (45 SP, 7 NIC) from the University of Mississippi from March 2014 to October 2015. Baseline characteristics at enrollment are presented in Table 1.

**Table 1:** Baseline demographics and clinical characteristics for non-infections disease controls and septic patients

Variables	NIC	SP	*p* value
	(*n* = 32)	(*n* = 109)	
Age, mean (SD)	62.71 (14.03)	60.87 (14.56)	0.53
Non-white, *n* (%)	9 (28.13)	31 (28.44)	0.95
Female, *n* (%)	10 (31.00)	43 (39.45)	0.47
Lactate (mg/dL), median (IQR)	1.2 (1–1.5)	2 (1.3–3.5)	0.04
PCT (ng/ml), median (IQR)	0 (0–0)	0 (0–4.122)	0.07
CitH3 (pg/ml), median (IQR)	34 (0–91.5)	101.5 (67–166)	< 0.0001
Survivors, *n* (%)	29 (90.63)	74 (67.89)	0.01
Mechanical ventilation, *n* (%)	0 (0)	24 (22.02)	< 0.0001
Length of hospital stay (d), median (IQR)	1.5 (1–3.5)	7.5 (4–12)	< 0.001
Total SOFA, median (IQR)	1 (0–3)	6 (4–10)	< 0.0001
SOFA components			
Respiratory, median (IQR)	0 (0–3)	1 (0–3)	< 0.0001
Renal, median (IQR)	0.5 (0–1)	1 (0–2)	0.16
Cardiovascular, median (IQR)	0 (0–0)	1 (0–4)	< 0.0001
Neurologic, median (IQR)	0 (0–0)	1 (0–2)	< 0.0001
Hepatic, median (IQR)	0 (0–0)	0 (0–0.25)	0.12
Coagulation, median (IQR)	0 (0–0)	1 (0–2)	< 0.0001

Categorical variables are presented as number (%). For continuous variables, normally distributed are presented as mean (SD), otherwise shown as median (interquartile range)

*SOFA* sequential organ failure assessment, *IQR* interquartile range, *CitH3* citrullinated histone H3, *PCT* procalcitonin, *NIC* non-infections disease controls, *SP* septic patients

Overall, SP were significantly more severely ill than NIC as evidenced by higher lactate levels [median (interquartile range, IQR) 2 mg/dL (1.3–3.5) vs 1.2 mg/dL (1–1.5), *p* = 0.04], higher need for mechanical ventilation [number (percentage) 24 (22.02%) vs 0 (0%), *p* < 0.0001], longer hospital stays [median (IQR) 7.5d (4–12) vs 1.5d (1–3.5), *p* < 0.001], lower 90-day survival rates [number (percentage) 74 (67.89) vs 29 (90.63%), *p* = 0.01], and higher SOFA scores [median (IQR) 6 (4–10) vs 1 (0–3), *p* < 0.0001]. Drivers of the difference in total SOFA score included respiratory, cardiovascular, neurologic, and coagulation components.

### Sub-group analysis

Results of the sub-group analysis in the UMich cohort are reported in detail in Supplemental Table 1. In brief, analysis of additional clinical parameters revealed that white blood cell and absolute neutrophil counts were not different between the SP and NIC groups (*p* = 0.29 and 0.07, respectively), even though the septic group contained a higher percentage of patients on immunosuppressive agents (53.03% vs 21.74% in SP vs NIC, respectively, *p* = 0.01). Body temperature was higher in SP (*p* = 0.03). CitH3 levels did not correlate with immunosuppression, body temperature, white blood cell or absolute neutrophil counts.

A source of infection was identified in 45 (68.33%) of the SP, with the respiratory system as the most common source of infection (Supplemental Table 2). Among those patients with an identified source of septic shock, there were 31 patients with available microbial culture data. Results of the cultures included: 1 patient with disseminated fungal infection (CitH3 at enrollment 348 pg/ml), 3 with positive viral cultures (CitH3 at enrollment: 0, 30, 108 pg/ml), and 27 with bacterial infections of varying types (Supplemental Table 2) [Median (IQR) CitH3 106 pg/ml (54–172) at enrollment]. Hypovolemic and cardiogenic shock were the most common presentations of non-infectious shock in the NIC group (Supplemental Fig. 2).

### Level of CitH3 in serum

**Fig. 1:**
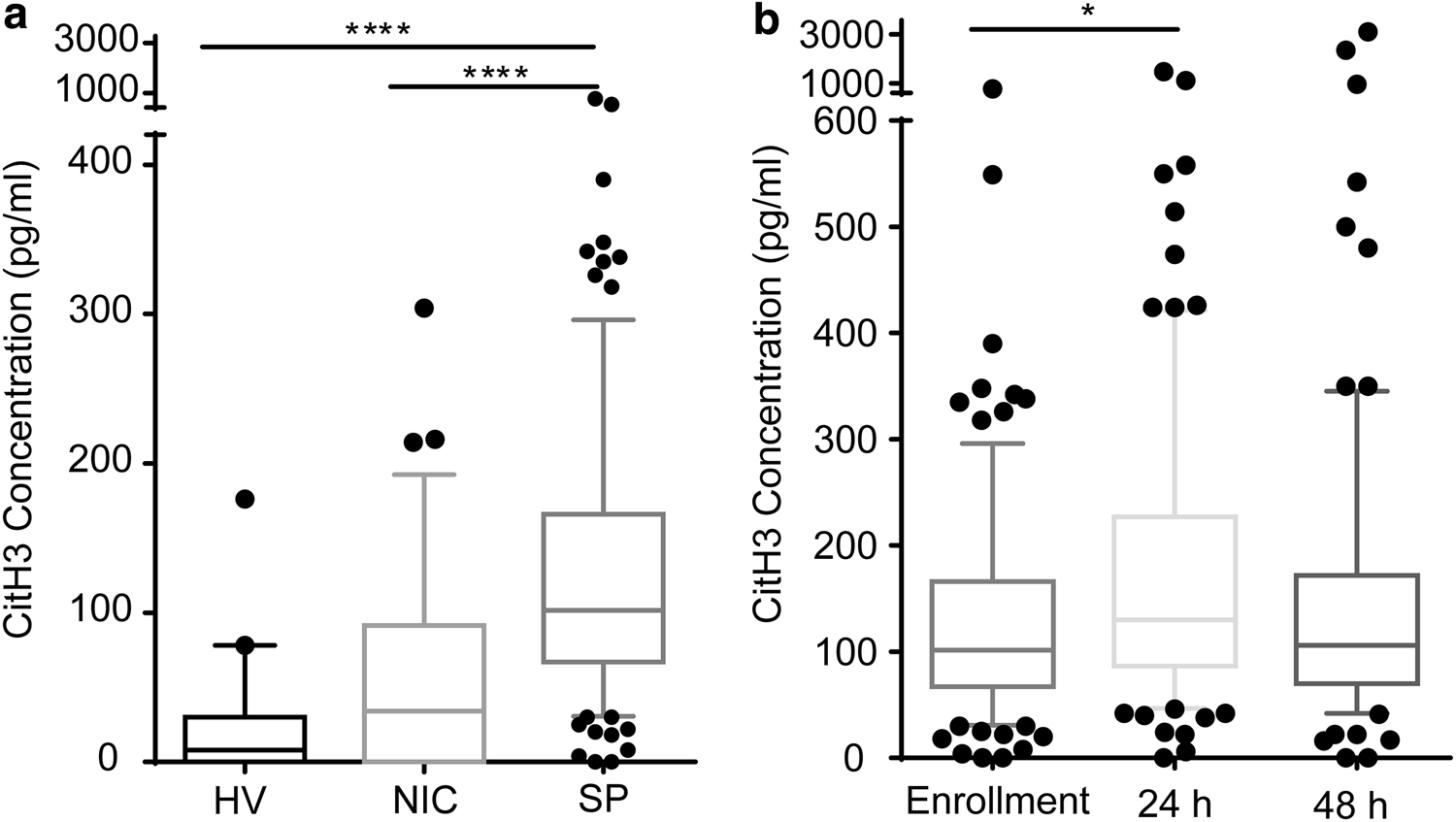
Citrullinated histone H3 is elevated in septic patients. **a** Citrullinated histone H3 in healthy volunteers, non-infections disease controls and septic patients at the enrollment in the emergency department. Citrullinated histone H3 was higher in septic patients than healthy volunteers and non-infections disease controls. **b** Citrullinated histone H3 levels in septic patients over time. Citrullinated histone H3 increased during the first 24 h but not 48 h. Data are presented as median value (line in box), interquartile range (box) and 90% (whiskers). Kruskal–Wallis test followed by Dunn’s multiple comparison test was performed for difference analyses. Linear mixed effects regression was further analyzed in Fig. 1b. *p < 0.05, ****p < 0.001. *HV* healthy volunteers, *NIC* non-infections disease controls, *SP* septic patients, *CitH3* Citrullinated histone H3

CitH3 was significantly elevated in SP compared to both HV and NIC at enrollment [median (IQR): SP 101.5 pg/ml (67–166); HV 8 pg/ml (0–30), *p *< 0.0001; NIC 34 pg/ml (0–91.5), *p* < 0.0001], there was no significant difference between HV and NIC (*p *= 0.20) (Fig. 1a).

Findings from the linear mixed models revealed there was a significant effect of time on CitH3 levels such that the CitH3 level at 24 h was significantly higher than the enrollment levels (*b* = 0.266; se = 0.116; *p* = 0.001). However, the CitH3 level at 48 h was not significantly different from the baseline levels (Fig. 1b).

### CitH3 and diagnosis

**Fig. 2:**
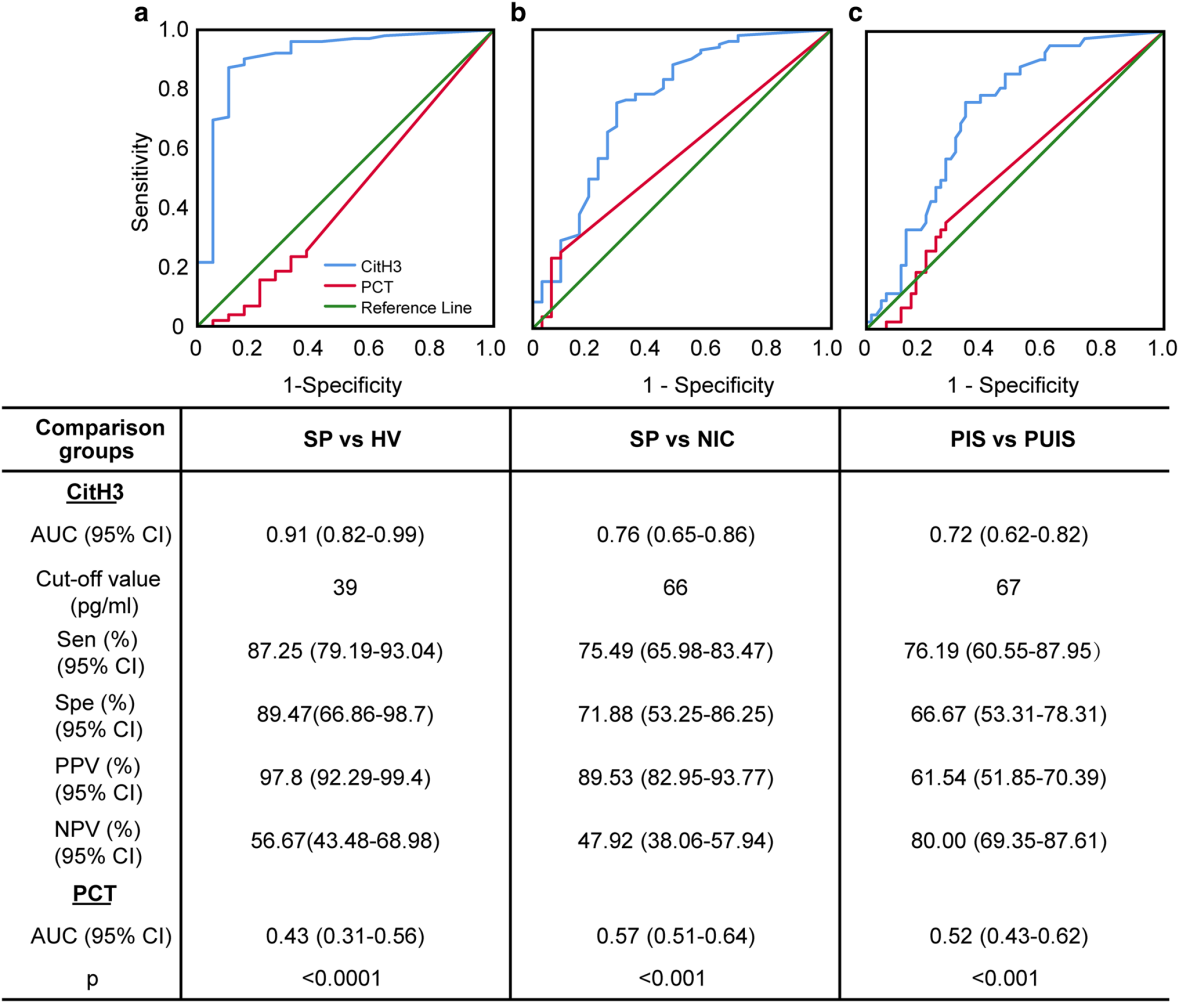
Citrullinated histone H3 performs better as a candidate diagnostic biomarker than procalcitonin for sepsis. Receiver operating characteristic curves analysis of citrullinated histone H3 and procalcitonin for sepsis diagnosis compared to (**a**) healthy controls and (**b**) non-infections disease controls. **c** Receiver operating characteristic curve analysis of citrullinated histone H3 and procalcitonin to distinguish septic patients with identified infection source from septic patients with un-identified infectious source. The receiver operating characteristic curve analysis of citrullinated histone H3 and procalcitonin are presented in the separate table under the figures. The values of *p* < 0.05 for CitH3 in all receiver operating characteristic curves and *p*  > 0.05 for procalcitonin. The *p* value in the last row of the table shows the difference of area under the receiver operating characteristic curves between citrullinated histone H3 and procalcitonin. *AUC* area under the receiver operating characteristic curve, *CI* confidence interval, *CitH3* citrullinated histone H3, *PCT* procalcitonin, *PPV* positive predictive value, *NPV* negative predictive value, *SP* septic patients, *HV* healthy volunteers, *NIC* non-infections disease controls, *PIS* septic patients with identified infection source, *PUIS* septic patients with un-identified infection source.

The diagnostic value of CitH3 was evaluated in comparison to PCT. There was no significant difference in PCT concentration between any of the groups in our study **(**Supplemental Fig. 3a), and its concentration did not vary significantly over time in SP (Supplemental Fig. 3b). CitH3 showed better diagnostic power than PCT on all ROC curves. AUCs (95% CI) for CitH3 and PCT to differentiate septic shock from a healthy state were 0.91 (0.82–0.99) vs 0.43 (0.31–0.56), respectively (*p* < 0.0001); and to distinguish septic from non-septic shock was 0.76 (0.65–0.86) vs 0.57 (0.51–0.64), respectively (*p* < 0.001) (Fig. 2a ,b). On sub-group analysis, CitH3 level was better able to distinguish SP with a clearly identified source of infection (PIS) from those without (PUIS); the AUCs of CitH3 and PCT were 0.72 (0.62–0.82) vs 0.52 (0.43–0.62), *p* < 0.001 (Fig. 2c).

A CitH3 level above 39 pg/mL was highly predictive of patients with septic shock compared to healthy volunteers (Fig. 2a; 97.8% positive predictive value, PPV). A presenting CitH3 level above 66 pg/ml distinguished SP from NIC with a PPV of 89.5% (Fig. 2b). On sub-group analysis, a CitH3 level less than 67 pg/ml was highly predictive of patients that later proved to have no identified source of microbial infection (80% negative predictive value, NPV; Fig. 2c).

### CitH3 and prognosis

**Fig. 3:**
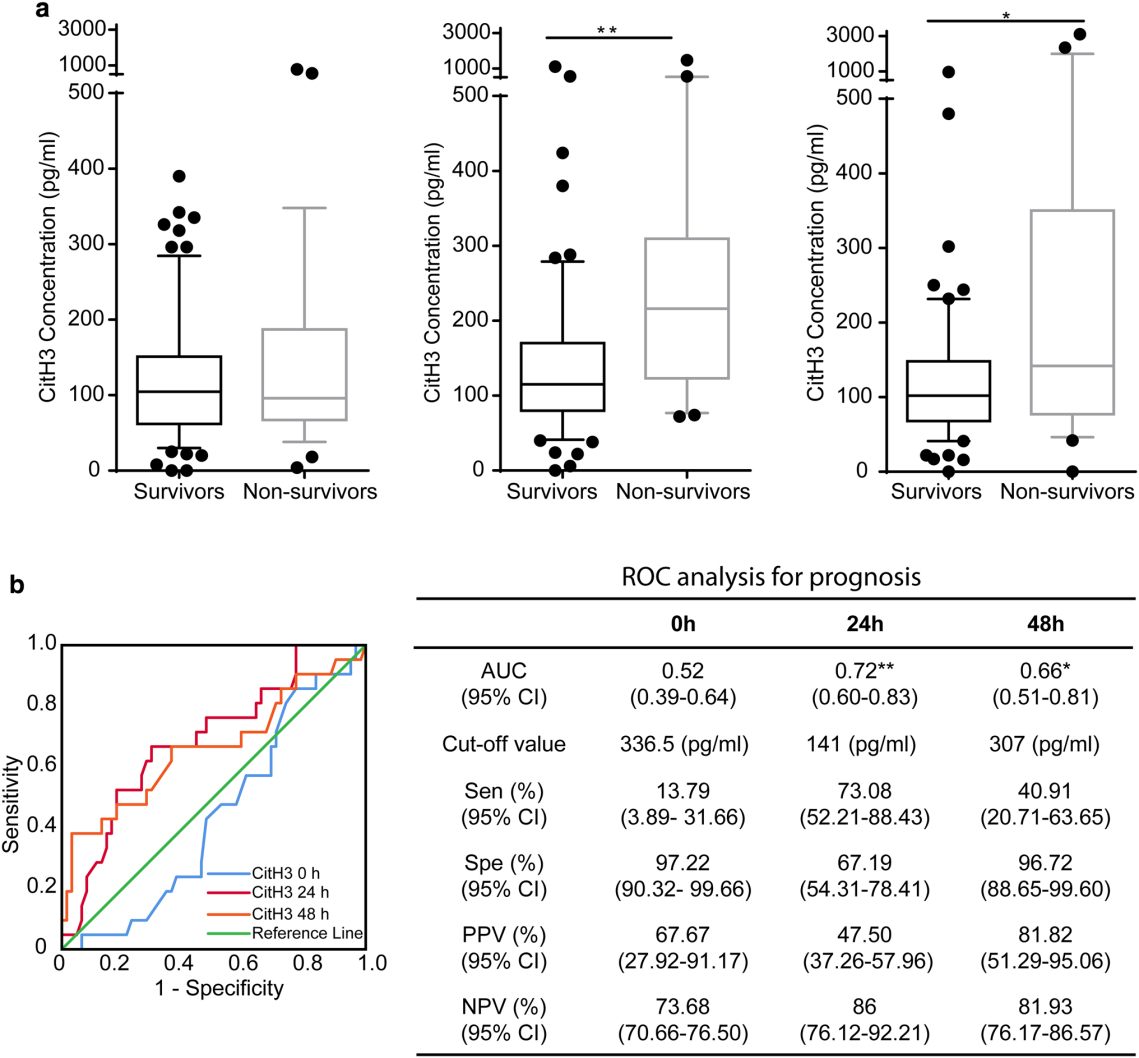
Citrullinated histone H3 is a potential prognostic marker in advanced sepsis. **a** Citrullinated histone H3 levels in sepsis 90d survivor and non-survivor groups at enrollment (0 h), 24 h and 48 h. Levels of citrullinated histone H3 were significantly elevated in the non-survivor group than survivor group at 24 h and 48 h, not at the enrollment (0 h). Data are presented as median value (line in box), interquartile range (box) and 90% (whiskers). **b** Receiver operating characteristic curves of citrullinated histone H3 to discriminate between survivors and non-survivors at enrollment, 24 h and 48 h. The receiver operating characteristic curve analysis of citrullinated histone H3 is presented in the separate table next to the figure. The area under the receiver operating characteristic curves are **p < 0.01 and *p < 0.05 at 24 h and 48 h, and *p* > 0.05 at enrollment (0 h). *ROC* receiver operating characteristic curve, *Sen* sensitivity, *Spe* specificity, *PPV* positive predictive value, *NPV* negative predictive value; *CitH3* citrullinated histone H3

While the CitH3 levels at the time of initial presentation were not significantly different between patients who survived and those who did not, the concentrations of CitH3 at 24 h (*p* < 0.01) and 48 h (*p* < 0.05) were significantly higher in SP who died within 90 days compared to those who survived (Fig. 3a). This finding is further supported by the ROC analysis that showed the AUCs (95% CI) for mortality were statistically significant at 24 h [0.72 (0.60–0.83)] and 48 h [0.66 (0.51–0.81)], but not at enrollment 0 h [0.52 (0.39–0.64)] (Fig. 3b). Furthermore, a CitH3 level above 307 pg/mL at 48 h was 96.72% specific for death within 90 days (Fig. 3b).

### Association between CitH3 levels and disease severity

**Fig. 4:**
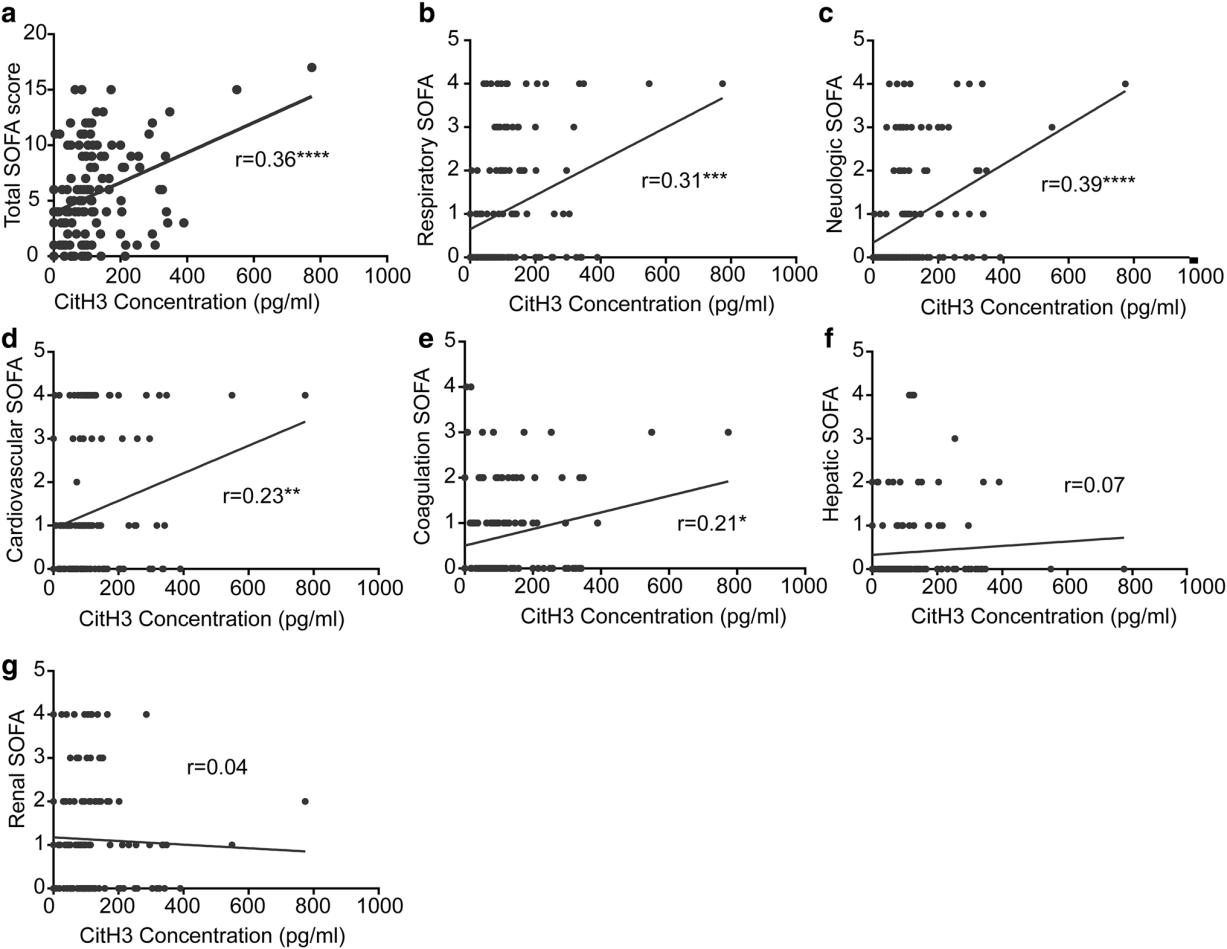
Serum citrullinated histone H3 concentration is positively correlated with sequential organ failure assessment (SOFA) score. Association between citrullinated histone H3 level and (**a**) total and (**b–g**) individual component SOFA scores. Pearson regression of citrullinated histone H3 and SOFA score is shown as a black line. CitH3, citrullinated histone H3

There was a positive correlation between CitH3 level and SOFA score (*r* value = 0.36, *p* < 0.0001, Fig. 4a). Analysis of SOFA score components revealed that CitH3 correlated with the respiratory, cardiovascular, neurologic, and coagulation component scores (Fig. 4b–g). There was no significant correlation between CitH3 and serum lactate levels (*p* = 0.09) (Supplemental Fig. 4).

### Correlation of CitH3 with PAD2/PAD4 in serum

**Fig. 5:**
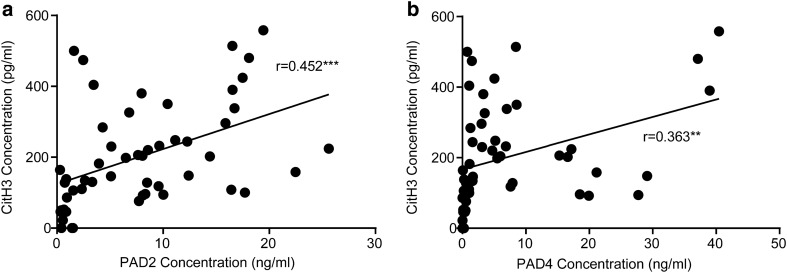
Association between citrullinated histone H3 level and PAD2 and PAD4 in septic patients. Pearson regressions of citrullinated histone H3 and PAD2 and PAD4 in septic patients are shown as a black line (*n* = 52). *PAD* peptidylarginine deiminase. **p < 0.01 and ***p < 0.001

There were positive correlations between circulating CitH3 and both PAD2 (r value = 0.452, p < 0.001) and PAD4 (*r* value = 0.363, *p* < 0.01) in septic shock patients (Fig. 5).

## Discussion

Early diagnosis of microbial infections and the initiation of appropriate antibiotic therapy are essential steps in improving clinical outcomes in septic patients [1]. Understanding the molecular mechanisms underlying the pathogenesis of sepsis is an important next step for developing novel diagnostic and treatment modalities. The goal of this pilot study was to determine if the relationships of CitH3, PAD2, and PAD4 identified in murine NETosis were conserved in humans with microbial infections. We then sought to determine if circulating CitH3 was elevated in patients with septic shock, as it has been in rodent models of septic shock [10], and if higher CitH3 levels could indicate more severe illness. We identified that serum concentrations of CitH3 were positively correlated with PAD2 and PAD4 concentrations, consistent with preclinical studies. We then found that the CitH3 level at the time of initial ED presentation was significantly elevated in the serum of patients with septic shock compared, not only to healthy volunteers, but also to those with other forms of shock. The degree of CitH3 elevation correlated with disease severity, and persistently elevated CitH3 48 h after the ED admission was a predictor of death. Therefore, CitH3 may represent a key component in the pathophysiology of septic shock and may be a useful marker of systemic infection.

Preclinical mechanistic studies demonstrate CitH3, catalyzed by PAD2 and PAD4 [15, 17], is an important mediator of the immune response to sepsis. CitH3 is a potentiator of NETosis and inflammation; interrupting this positive feedback loop has improved survival in sepsis in mice [12]. In this study, we found that PAD2 has a relative higher r value (0.452 vs 0.363) with CitH3 than PAD4. This may partially explain why targeting PAD2 instead of PAD4 shows better protection in mouse models of sepsis and endotoxic shock [18, 19]. The positive correlation between circulating levels of CitH3, PAD2, and PAD4 in patients with septic shock confirms their potential connection in the pathogenesis of sepsis. Given the inflammatory nature of CitH3, blocking it or PAD2 may also provide a new potential treatment targets for septic shock.

Studies from other investigators have unveiled CitH3 as potential diagnostic and prognostic blood marker associated with an exacerbated inflammatory response in patients with advanced cancer [20] and elevated in serval diseases like pneumonia, sepsis, and experimental endotoxemia [21–23]. Recently, Zuo et.al found that serum levels of CitH3 were increased in coronavirus disease 2019 (COVID-19) patients [24]. In this study, elevated CitH3 at presentation corresponded to the diagnosis of septic shock and the presence of an identified source of microbial infection. This finding is consistent with rodent models of endotoxic and septic shock that revealed CitH3 is released during the activation of host bacterial defenses [10, 12, 25]. Thus, our finding that healthy people had lower CitH3 levels than septic shock patients was consistent with expectations. We found that CitH3 concentrations exceeding 39 pg/ml had a positive predictive value approaching 98% for distinguishing SP from healthy individuals. Only patients with neutropenia (absolute neutrophil count below 1000/µL) or chronic polymicrobial urinary tract colonization exhibited serum CitH3 below 39 pg/ml in the presence of a positive bacterial culture. This finding may suggest reduced immune cell activation in these patients. In contrast to PCT that was not statistically different between SP and healthy individuals, CitH3 elevation was a specific indicator of infection.

While CitH3 may be a marker of neutrophil activation, some of the patients with neutropenia had elevated CitH3. This fact may be explained by CitH3 being produced outside of NETosis. The release of CitH3 by macrophages and monocytes has been documented in sepsis-induced METosis [26]. The additional sources of CitH3 may explain why serum CitH3 levels were not correlated with absolute neutrophil count (Supplemental Table 1). There may be a critical threshold for neutrophil or monocyte count below which CitH3 cannot be adequately produced in response to a bacterial infection, rendering it less reliable in patients with severe neutropenia and leukopenia. Since distinguishing patients in septic shock from healthy people does not typically require a laboratory test, identifying low CitH3 levels in non-neutropenic patients suspected of having sepsis may have more utility. This cohort may benefit from a broader infectious workup including identifying sources of viral illness and chronic infection. More research will be needed to confirm these findings with a larger population of these patients.

High levels of CitH3 may be useful to identify patients that could have an occult infection. A serum CitH3 level of 66 pg/ml or greater was able to identify patients with microbial infection with reasonable sensitivity and specificity. Of the three NIC patients that had CitH3 above 66 pg/ml, two had been discharged less than 7 days prior following a separate hospitalization for sepsis and the remaining patient was later found to have endocarditis. Therefore, in patients that are not initially suspected of having sepsis, elevated CitH3 may give support to starting empiric antibiotics and broadening the infectious workup.

High levels of serum CitH3 were found in patients with more severe infection, which is in keeping with preclinical data that identified a positive feedback loop that progressively increases CitH3 levels in the presence of sepsis. The median concentration of serum CitH3 in the SP group was over 100 pg/ml, nearly triple the median CitH3 concentration of the NIC patients in shock (Fig. 1a). Patients with higher SOFA scores exhibited a positive, linear correlation with serum CitH3 concentration (Fig. 4). Those with high CitH3 levels 24 h and 48 h after the initiation of treatment were high risk of early mortality. Interestingly, some of the highest CitH3 levels were observed in patients with fungemia, which may represent enhanced NETosis or METosis in response to disseminated fungal infections. These finding may indicate the presence of uncontrolled infection, inadequate antimicrobial therapy, or a maladaptive inflammatory response in patients with persistently elevated CitH3 (Fig. 3a). Clinically, these patients may warrant broader antibiotics, the addition of anti-fungal agents, a wider diagnostic work up, and/or a discussion regarding goals care.

This study has several limitations. Because it is a proof-of-concept study that involved analysis of blood samples and clinical data collected as part of a separate trial, we did not have access to all the clinical details from the cohort enrolled at the University of Mississippi. This further limited the sample size, which reduced the power of our sub-group analysis. CitH3 levels were only followed for the first 48 h after the ED admission. The trajectory of CitH3 levels over time may be informative of a patient’s response to therapy and clinical course. Tracking the trajectory of CitH3 over longer periods may reveal how long it remains elevated following the clinical resolution of sepsis. Additionally, the patients in this study were all severely ill with evidence of shock. Future studies are needed to explore the role of CitH3 in the progression of sepsis to septic shock and to determine whether this pathway could lead to new therapeutic targets.

## Conclusions

This proof-of-concept study demonstrated that CitH3 is increased in patients with septic shock and positively correlated with PAD2 and PAD4. This finding suggests that human patients may share the PAD-CitH3 pathway identified in preclinical models and implicated in the development of the clinical manifestations of sepsis. The serum concentration of CitH3 correlates with the severity of organ injury and prognosis. CitH3 may provide insight into the pathophysiology of septic shock and aid in the development of new therapeutics. Further investigations are required to evaluate CitH3 patterns in sepsis compared to other conditions that activate the innate immune system. Understanding these molecular mechanisms in the pathogenesis of sepsis will be an important next step for developing novel diagnostic and treatment modalities.

## Electronic supplementary material

Below is the link to the electronic supplementary material.

Supplementary file1 (DOCX 339 kb)

## Abbreviations

AUC: Area under the curve
CI: Confidence interval
CitH3: Citrullinated histone H3
CLP: Cecum ligation and puncture
DAMP: Damage-associated molecular pattern
ED: Emergency department
ELISA: Enzyme-linked immunosorbent assay
FiO2: Fraction of inspired oxygen
ICU: Intensive care unit
IL-1β: Interleukin 1 beta
LPS: Lipopolysaccharides
HV: Healthy volunteers
mAb: Monoclonal antibody
METosis: Macrophage/monocyte extracellular traps (programmed cell death)
NETs: Neutrophil extracellular traps
NETosis: Neutrophil extracellular traps (programmed cell death)
NIC: Non-infection disease controls
NPV: Negative predictive value
PaO2: Partial pressure of oxygen in arterial blood
PCT: Procalcitonin
PAD2: Peptidyl arginine deiminase 2
PAD4: Peptidyl arginine deiminase 4
PPV: Positive predictive value
SOFA: Sequential organ failure assessment
SpO2: Oxygen saturation
TNF-α: Tumor necrosis factor-alpha
