# Supplementary material for: Serum citrullinated histone H3 concentrations differentiate patients with septic verses non-septic shock and correlate with disease severity
Source: Infection. 2020 Sep 30;49(1):83–93. doi: 10.1007/s15010-020-01528-y (PMC7527151; doi:10.1007/s15010-020-01528-y)
Supplement: Supplementary file 1 — Supplementary file1 (DOCX 339 kb) [file 15010_2020_1528_MOESM1_ESM.docx]

**Supplemental Material****s**

**Title:** Serum Citrullinated Histone H3 Concentrations Differentiate Patients with Septic Verses Non-septic Shock and Correlates with Disease Severity

**Journal:** Infection

**Authors:** Yuzi Tian, MD1, 2 #; Russo Rachel, MD1#; Yongqing Li, MD, PhD1*; Monita Karmakar, PhD1; Baoling Liu, MD1; Michael A. Puskarich3, 4, MD; Alan E. Jones, MD^5^; Kathleen A. Stringer, PharmD6, 7; Theodore J. Standiford, MD7; Hasan B. Alam, MD, FACS1*

# Affiliations:

1 Department of Surgery, University of Michigan Health System, Ann Arbor, Michigan, USA.

2 Department of Rheumatology, Xiangya Hospital, Central South University, Changsha, Hunan, China.

^3^ Department of Emergency Medicine, Hennepin County Medical Center, Minneapolis, Minnesota, USA.

^4^ Department of Emergency Medicine, University of Minnesota, Minneapolis, Minnesota, USA.

^5^ Department of Emergency Medicine, University of Mississippi Medical Center, Jackson, Mississippi, USA.

^6^ Department of Clinical Pharmacy, College of Pharmacy, University of Michigan, Ann Arbor, Michigan, USA.

^7^ Division of Pulmonary and Critical Care Medicine, Department of Internal Medicine, University of Michigan School of Medicine, Ann Arbor, Michigan, USA.

This work was performed at University of Michigan.

**#**These authors contributed equally to this work.

# * Correspondence:

Yongqing Li, MD, PhD: yqli@med.umich.edu

Hasan B. Alam, MD: [alamh@med.umich.edu](mailto:alamh@med.umich.edu)

**Supplemental Table 1** Additional clinical information for patients enrolled at University of Michigan and its correlation with CitH3

| **Variable** | **NIC** | **SP** | **P**  **values** | **Correlation with CitH3** |
| --- | --- | --- | --- | --- |
| Not immunosuppressed | 18, (78.26%) | 31, (46.97%) | 0.01 | NS |
| Immunosuppressed | 5, (21.74%) | 35, (53.03%) |  |  |
| Temperature (℃) | 17, 36.6 (36.5-36.8) | 50, 36.9 (36.5-37.5) | 0.03 | NS |
| WBC (K/μl) | 23, 8.7(6.6-13.1) | 58, 11.0 (6.4-14.7) | 0.29 | NS |
| Neutrophil counts (K/μl) | 23, 6.82 (4-8.2) | 57, 10.12 (5.2-12.5) | 0.07 | NS |

Categorical variables are presented as number (%). For non-normally distributed continuous variables, results are shown as number of patients, median (interquartile range). NIC, non- infections disease controls; SP, septic patients; NS: no significance; WBC, white blood cell.

**Supplemental Table 2** Additional infection information for septic shock patients enrolled at University of Michigan

| **Infection source** |  |
| --- | --- |
| Respiratory | 16 |
| Urinary | 11 |
| Abdominal | 10 |
| Skin and soft tissues | 5 |
| Cardiac | 2 |
| Central nervous system | 1 |
| Unknown | 19 |
| **Culture organisms** |  |
| *Escherichia coli* | 5 |
| *Clostridium difficile* | 3 |
| *Methicillin-resistant Staphylococcus aureus* | 3 |
| *Vancomycin-resistant Enterococci* | 2 |
| *Klebsiella pneumoniae* | 2 |
| *Streptococcus pneumoniae* | 1 |
| *Staphylococcus epidermidis* | 1 |
| *Proteus mirabilis* | 1 |
| Multiple organisms | 9 |
| Bacterial culture negative | 33 |


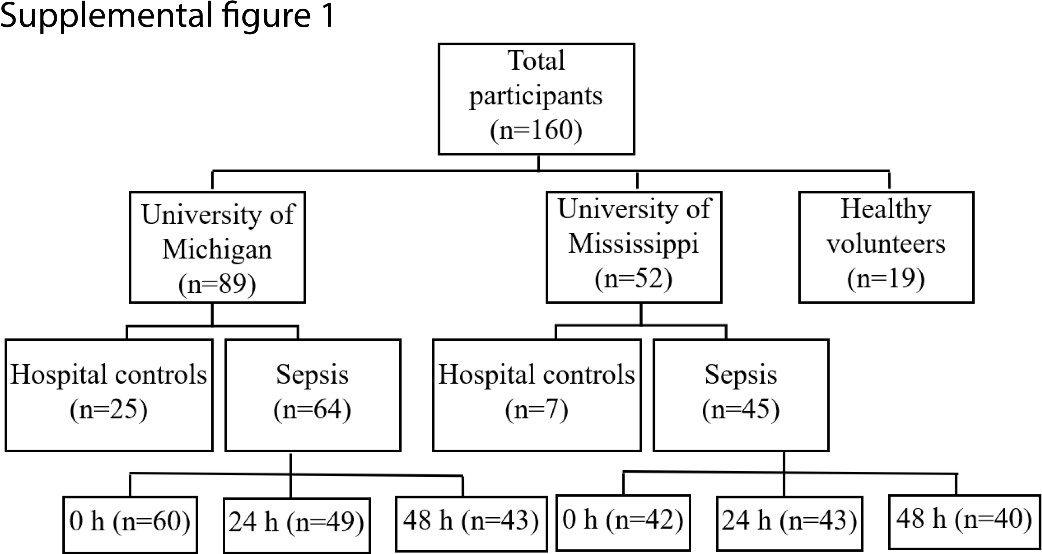


**Supplemental Fig. 1.** Number of subjects and time points for each cohort and overall.


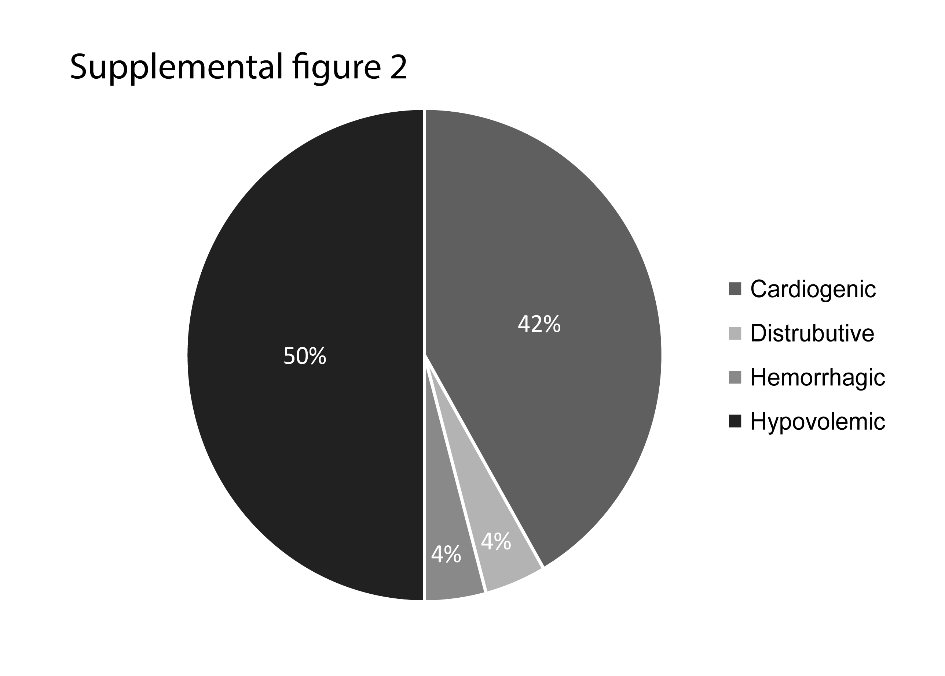


**Supplemental Fig. 2**. Types of Non-infectious shock within the non-infectious control group.


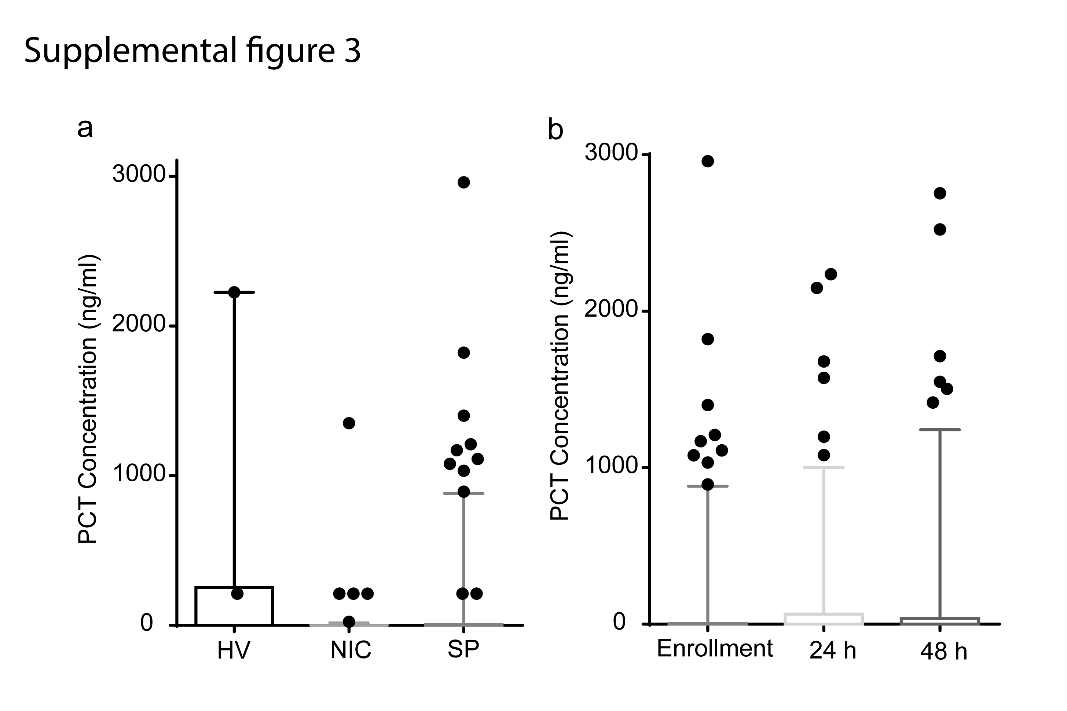


**Supplemental Fig. 3.** Levels of procalcitonin in septic patients. (a) Procalcitonin levels in healthy volunteers, non-infections disease controls and septic patients at the enrollment in the emergency department. Procalcitonin didn’t change among three groups. (b) Procalcitonin levels in septic patients over time. Procalcitonin did not change over time. Data are presented as median value (line in box), interquartile range (box) and 90% (whiskers). Kruskal-Wallis test followed by Bonferroni’s multiple comparison test was performed for difference analyses. Linear mixed effects regression was further analyzed in Figure b. HV, healthy volunteers; NIC, non-infections disease controls; SP, septic patients.


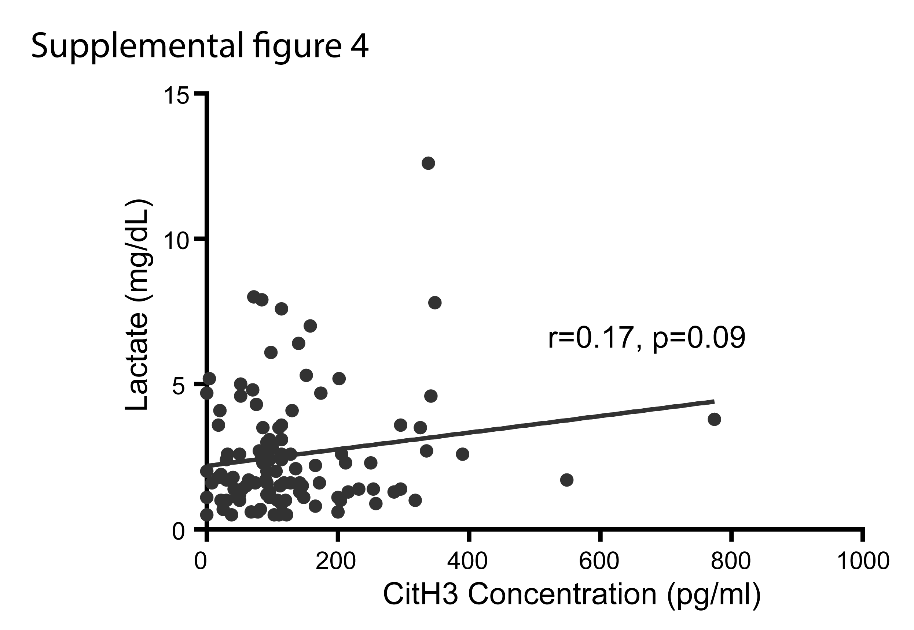


**Supplemental Fig. 4.** Association between citrullinated histone H3 level and lactate. Pearson regression of citrullinated histone H3 and lactate is shown as a black line. CitH3, citrullinated histone H3.
